# Influence of Contact Stress on Surface Microstructure and Wear Property of D2/U71Mn Wheel-Rail Material

**DOI:** 10.3390/ma12193268

**Published:** 2019-10-08

**Authors:** Chun-Peng Liu, Xiu-Juan Zhao, Peng-Tao Liu, Jin-Zhi Pan, Rui-Ming Ren

**Affiliations:** 1 School of Material Science and Engineering, Dalian Jiaotong University, Dalian 116028, China; liuchunpeng1991@sina.com (C.-P.L.); zhaoxj@djtu.edu.cn (X.-J.Z.); liupptt@163.com (P.-T.L.); 2 Key Laboratory of Critical Materials of Rail Transportation in Liaoning Province, Dalian Jiaotong University, Dalian 116028, China; 3 National and Local Joint Engineering Center of Rail Transit Equipment Design and Manufacturing Technology, Dalian Jiaotong University, Dalian 116028, China; jinzhip@163.com

**Keywords:** D2 wheel steel, U71Mn rail steel, contact stress, surface microstructure, fatigue wear

## Abstract

To investigate the relationship between surface microstructure and wear mechanism in D2/U71Mn wheel-rail material under different contact stress conditions, rolling wear tests using a GPM-40 wear machine to simulate the wheel-rail operation was performed. After wear tests, an optical microscope (OM), scanning electron microscope (SEM) and micro-hardness testers were used to characterize the microstructure and fatigue wear cracks. The results show that the thickness of the plastic deformation layer and surface hardness is increased with the increase of contact stress. Under high contact stress condition (1200 MPa), the severe plastic deformation layer led to the formation of fatigue wear of wheel-rail samples. Under a contact stress of 700 MPa, the wear mechanism of samples is adhesive wear and wear rate is low. With the increase of contact stress, the fatigue cracks are gradually severe. Under a contact stress of 1200 MPa, the wear mechanism of samples becomes fatigue wear and the fatigue wear cracks cause the increase of wear rate. The fatigue wear can accelerate the wear failure of wheel-rail samples. The fatigue wear cracks of wheel samples are severer than that of rail samples due to both the rate of plastic strain and the content of proeutectoid ferrite.

## 1. Introduction

In recent years, high-speed trains become one of important passenger transportation all over the world. High-speed trains have the advantages of convenience, large transport capacity, safety and so on. However, the wear and rolling contact fatigue failure of the wheel-rail material are serious problems all the time. The severe wear and rolling contact fatigue of the wheel surface are not only causing economic loss but also rise of traffic accidents. As increase of the axle load of high-speed trains, the wear and fatigue failure of the wheel-rail material are more severe, such as rail corrugation and wheel polygonization wear [1,2], partial grinding of wheel, and the abrade of wheel tread [3]. Therefore, an investigation on the influence of contact stress on the evolution of the surface microstructure and the wear mechanism of the the wheel-rail material is greatly important to improve the service life of the wheel-rail material.

The wear failure of the wheel-rail material is a complex process. Many factors can change the wear property, such as the slip ratio, rolling speed and original microstructure, etc. Under the dry friction condition, the thickness of the plastic deformation layer is increased with the increase of the slip ratio. Simultaneously, the wear mechanism of wheel-rail materials transforms into fatigue wear, the shallow fatigue wear cracks are formed during the fatigue wear process [4,5,6]. The surface hardness and the thickness of the plastic deformation layer are gradually decreased as the rolling speed increases. The wear loss of the wheel material is increased, while that of the rail material is decreased with the rolling speed increasing [7]. With the increment of the axle load, the plastic deformation is more severe [8]. The different surface treatment methods also have an important influence on the wear loss of the wheel-rail materials [9,10]. Bolton and Clayton [11] proposed that there are three wear regimes during the wear process, which are the mild wear regime, severe wear regime and catastrophic wear regime. Moreover, Lewis [12] and Ding [13] also studied the wear regime of wheel-rail material during the wear process.

Different original microstructures have a diverse influence on wear performance of the wheel-rail material, for example, pearlite, bainite and martensite [14,15,16]. Zeng et al [17] indicated that the wear resistance and solution strength of the wheel-rail material are enhanced with the increase of carbon content. The wear resistance of lamellar pearlite is higher than that of spheroidal pearlite [18]. The wear resistance of U75V quenched rail is good, which is suitable for heavy haul railway. The fatigue resistance of U71Mn hot rolled rail steel is good. It is suitable for high-speed railway [8].

In the present paper, the D2 wheel steel is China’s own production of the wheel steel of high-speed trains. The U71Mn rail steel is the rail steel matched to the D2 wheel steel. The GPM-40 wear machine is used to simulate the operation of a wheel/rail system. The relationship between the evolution of the surface microstructure and wear mechanism of wheel-rail samples under different contact stress conditions is investigated. The influence of different wear mechanisms on the wear failure of wheel-rail samples is also discussed.

## 2. Material and Methods

**Figure 1:**
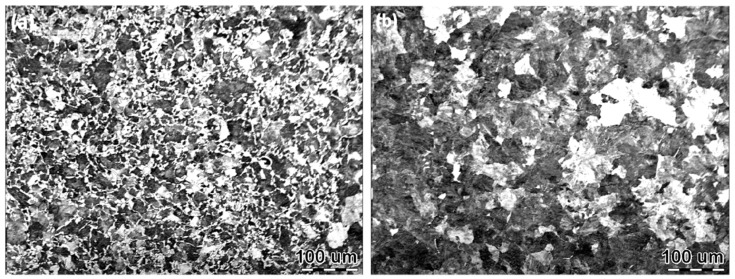
The original microstructure of wheel and rail samples. (**a**) D2 wheel steel and (**b**) U71Mn rail steel.

**Figure 2:**
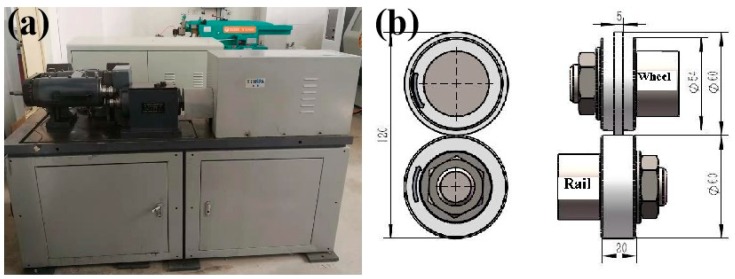
The test apparatus: (**a**) GPM-40 wear tester and (**b**) sample dimensions and contact mode of wheel and rail samples (unit: mm).

The chemical composition of D2 wheel steel and U71Mn rail steel is shown in Table 1. The original microstructure of D2 wheel steel and U71Mn rail steel are displayed in Figure 1. The original microstructure of the wheel samples was formed of pearlite and proeutectoid ferrite. The original microstructure of the rail samples was pearlite and a small amount of proeutectoid ferrite. The original hardness of the wheel samples was around 320 HV, and the yield strength and tensile strength of the wheel samples were 615 and 955 MPa, respectively. The rail samples had an original hardness of approximately 340 HV, the yield strength and tensile strength of the rail samples were 880 and 900 MPa, respectively. The wear tester (Yi Hua, Jinan, China) and wheel-rail sample dimensions are shown in Figure 2. The rolling of wheel-rail samples was realized by the rotation of two AC servo motors to drive the rotation of wheel-rail samples. The test force (F) was realized by a hydraulic actuator and hydraulic pump station. The contact stress values of wear tests were 700 MPa, 900 MPa and 1200 MPa. Three repeated wear tests were performed at each contact stress condition. The contact stress was calculated according to the Hertz contact theory [19]:

(1) $$P_{0}=0.418\sqrt{\frac{FE}{L}\left(\frac{1}{R_{w}}+\frac{1}{R_{r}}\right)}.$$

The *P_0_* is the contact stress, *F* is applying load, *E* is the modulus of the elasticity of steel, *L* is contact length and *R_w_* and *R_r_* are the radii of the wheel and rail sample. The rolling speed was 1440 r/min to simulate the operation speed of 250 km/h. The operating condition was the pure rolling operation without liquid lubrication. The operation cycle was 3 × 10^5^ cycles. During the rolling wear test, the samples were cooled by fans.

After the test, the surface macroscopic morphology of samples under different contact stress conditions was analyzed by a Universal Serial Bus microscope (Mustcam, Hong Kong, China). The surface microstructure of samples under different contact stress conditions was analyzed by using a Leica optical microscope (OM, Leica, Wetzlar, Germany) and Zeiss Supra 55 field-emission scanning electron microscope (SEM, Zeiss, Oberkochen, Germany). The surface hardness was measured by using a FM-700 hardness tester (Future-Tech, Kanagawa, Japan) with a load of 2.45 N and dwell time of 15 s.

## 3. Results 

### 3.1. Traction Coefficient

The traction coefficient μ is calculated by the following equation:

(2) $$\mu =M/F_{n}.$$

**Figure 3:**
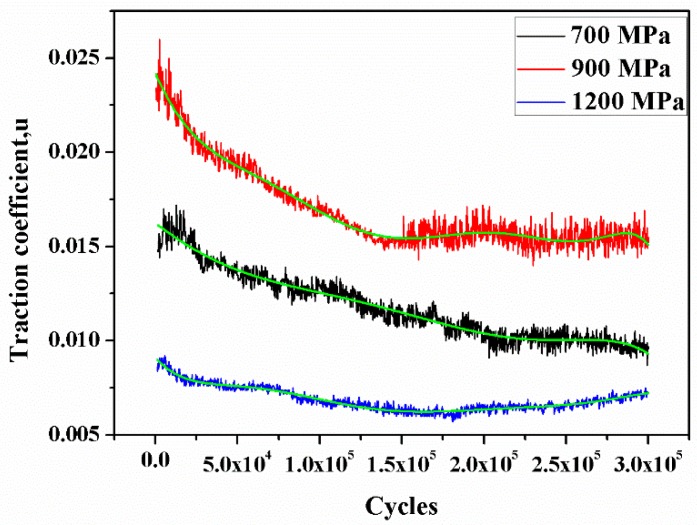
The variation of the traction coefficient under different contact stress conditions.

In equation, *M* is the traction torque and *F_n_* is the load. The traction coefficient under different contact stress conditions is displayed in Figure 3. All the curves are fitted by adopting six order polynomials fit of OriginPro 9.0. The curve fit equation is as follows:

(3) $$y=0.01624+B_{1}x+B_{2}x^{2}+B_{3}x^{3}+B_{4}x^{4}+B_{5}x^{5}+B_{6}x^{6}.$$

**Figure 4:**
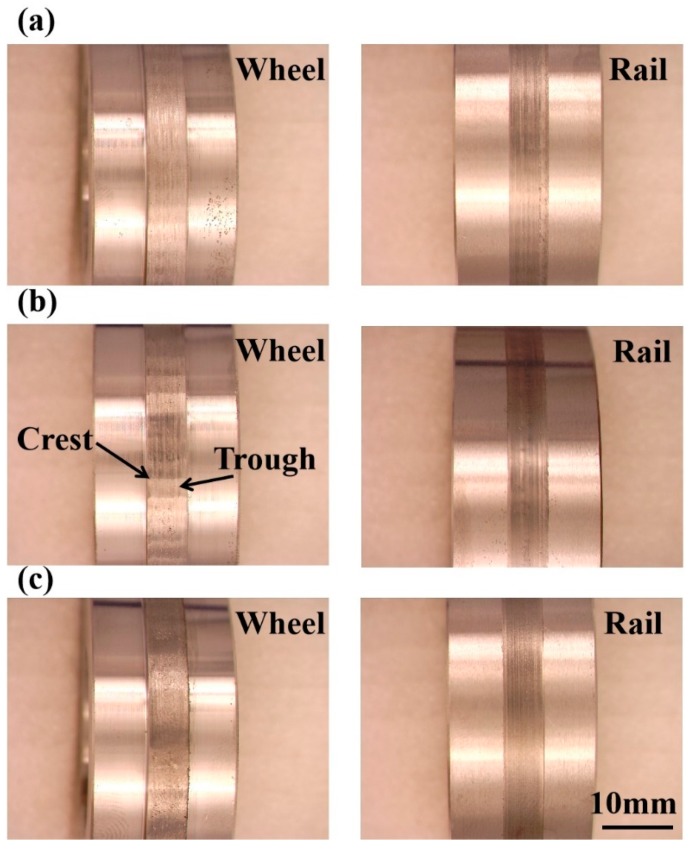
The surface macroscopic morphology of wheel and rail samples under different contact stress conditions: (**a**) 700 MPa; (**b**) 900 MPa and (**c**) 1200 MPa.

In the curve fit equation, B_1_ = −653721 × 10^−8^, B_2_ = 2.18025 × 10^−13^, B_3_ = −4.85653 × 10^−23^, B_4_ = −4.85653 × 10^−23^, B_5_ = 1.3424 × 10^−28^, B_6_ = −2.57054 × 10^−34^ and B_1_~B_6_ are the proportionality coefficients from OriginPro 9.0. It can be seen from Figure 3 that the variation of the traction coefficient under different contact stress conditions presented similar trends. At the initial wear stage, the traction coefficient rapidly increased. This is mainly because the contact area of wheel-rail samples was the point contact due to the existence of machining marks. The contact area was comparatively small during the initial wear stage. Therefore, the contact stress was relatively large and the traction coefficient was high. As the degree of wear increased, the contact area of the wheel-rail samples gradually was increased to cause the decrease of contact stress gradually. Moreover, the oxidative wear can also reduce the traction coefficient during the wear process [20]. Consequently, the traction coefficient gradually was decreased with the increase of cycles after the initial wear stage. Lastly, the traction coefficient was in the steady state. The variation of the traction coefficient during the rolling wear process was analogous to the result of Chen et al [21]. The traction coefficient value under a contact stress of 700 MPa was approximately 0.01, the surface of wheel-rail samples was relative to smooth (Figure 4a). Under the contact stress of 900 MPa, the traction coefficient value was at its maximum, which was about 0.016. The reason was that the surface of wheel samples form the polygonization wear (worn-surface of wheel samples form the crest and trough [2]), as shown in Figure 4b. The polygonization wear of wheel samples led to the contact surface of wheel-rail samples being in the rolling–sliding condition. The existence of the slip ratio will increase the shear stress of wheel-rail samples, which will cause an increase of traction coefficient. While the traction coefficient was the minimum under a contact stress of 1200 MPa. It was only about 0.008. The contact surface of wheel-rail samples was smooth and no polygonization wear was observed, as displayed in Figure 4c.

### 3.2. Surface Hardness and Wear Loss

**Figure 5:**
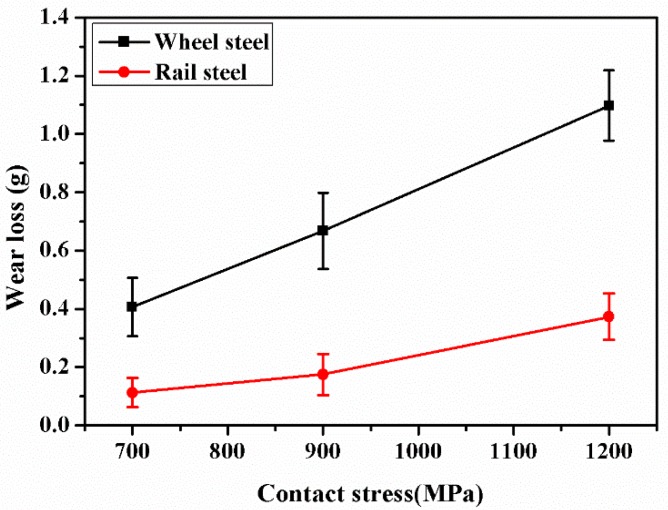
The variation of the wear loss of wheel and rail samples under different contact stress conditions.

Figure 5 shows the wear loss of wheel and rail samples under different contact stress conditions. As the contact stress increased, the wear loss of wheel-rail samples increased. However, the wear loss of wheel samples was remarkably higher than that of rail samples. Under 1200 MPa contact stress condition, the wear loss of wheel samples was five times higher than that of rail samples. The reasons should be that the carbon content of rail samples was higher than that of wheel samples, as seen in Table 1. The high carbon content could improve the matrix hardness, thus reducing the wear loss. The increase of carbon content could improve the wear resistance by enhancing the matrix hardness of materials [14].

**Figure 6:**
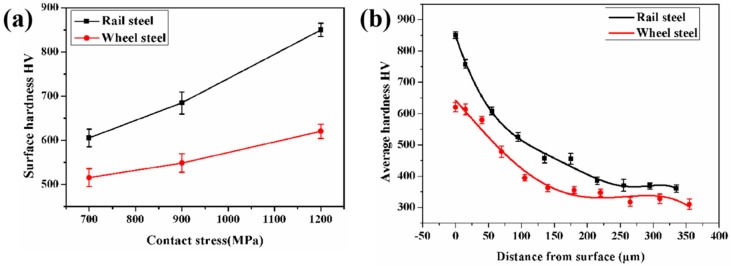
The variation of the hardness of wheel and rail samples. (**a**) Surface hardness under different contact stress conditions. (**b**) The hardness profile of wheel-rail samples from surface to matrix under a contact stress of 1200 MPa.

The variation of wear loss under different contact stress condition had a close relationship to the surface hardness of samples. Hence, the surface hardness of wheel-rail samples was measured, as shown in Figure 6. It is clearly shown from Figure 6a that the surface hardness of wheel-rail samples was increased as the contact stress increased, while the surface hardness of rail samples was higher than that of wheel samples. Figure 6b shows the variation of the hardness of wheel and rail samples as a function of the distance from the surface to undeformed matrix under a contact stress of 1200 MPa. The change of hardness of wheel-rail samples presented a similar tendency. The hardness dropped gradually from the surface to interior of samples. The surface hardness of the rail sample was 850 HV, which was higher than that of the wheel sample (620 HV). Moreover, the hardness of the rail sample was also higher than that of wheel sample at different positions from the surface. However, the thickness of the hardening layer of the wheel sample, which was about 270 μm, was larger than that of the rail sample (220 μm). This is due to the fact that the content of proeutectoid ferrite in the wheel sample was larger than that in the rail sample. It is of good ductility to contribute to a thicker hardening layer [4].

### 3.3. Surface Microstructure

**Figure 7:**
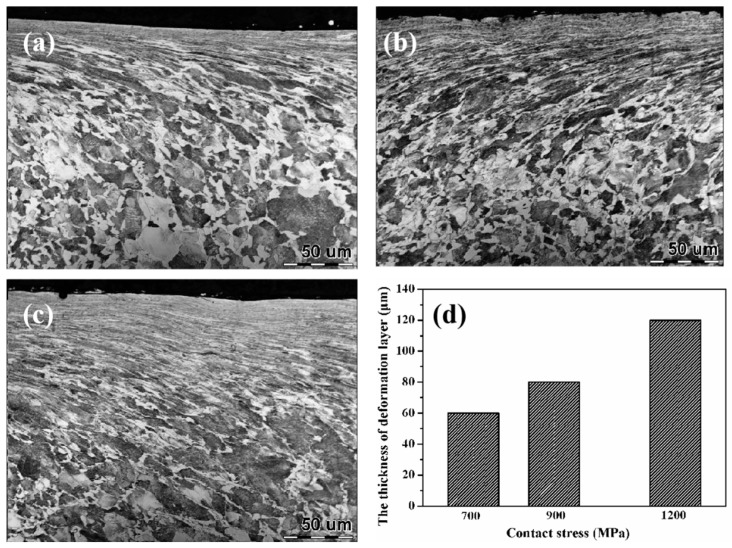
The optical microscope (OM) micrographs of wheel samples under different contact stress conditions (**a**) 700 MPa; (**b**) 900MPa; (**c**) 1200MPa and (**d**) the thickness of the plastic deformation layer.

**Figure 8:**
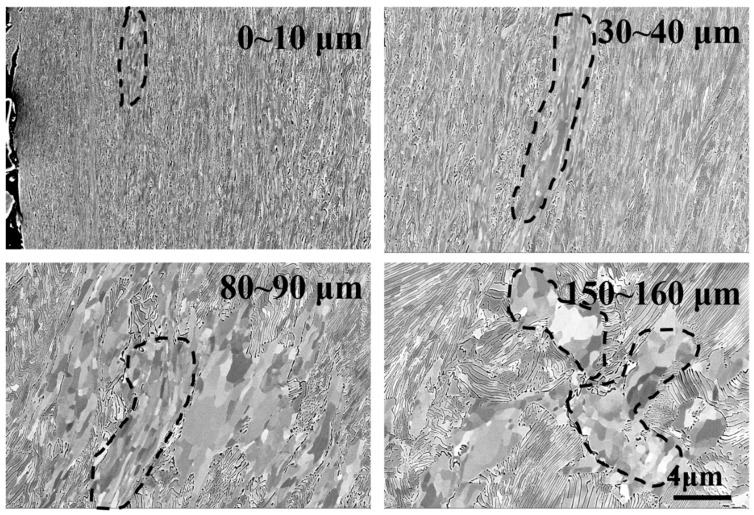
The SEM micrograph of wheel samples at different positions from the surface under a contact stress of 1200 MPa.

**Figure 9:**
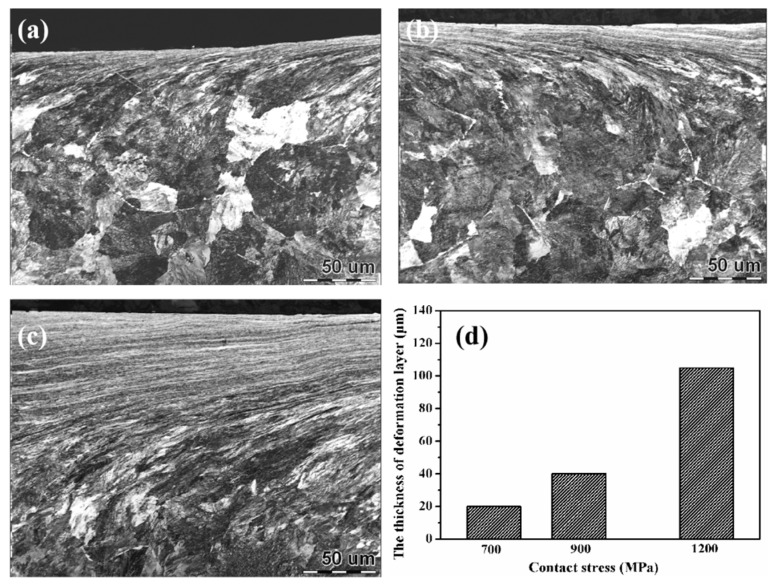
The OM micrographs of rail samples under different contact stress conditions: (**a**) 700 MPa; (**b**) 900 MPa; (**c**) 1200 MPa and (**d**) the thickness of the the plastic deformation layer.

**Figure 10:**
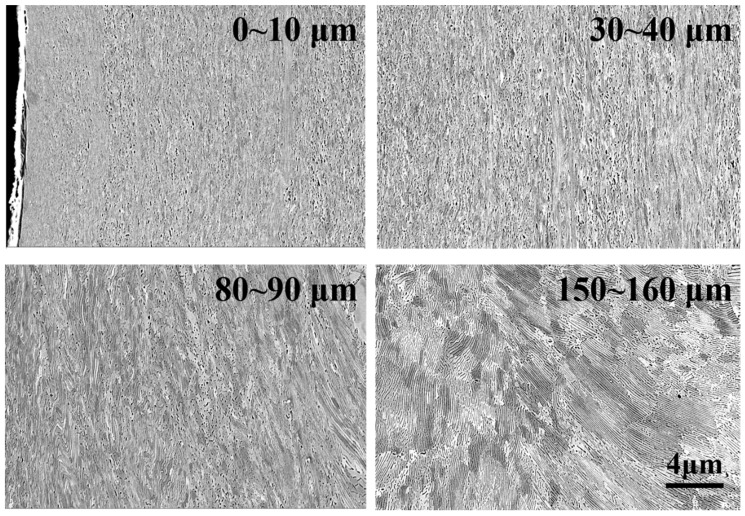
The SEM micrograph of rail samples from different positions in the top surface under contact stress of 1200 MPa.

During the rolling wear process, the surface of wheel-rail samples will form a certain thickness of plastic deformation layer rapidly [2,22]. The plastic deformation layer not only can change the surface hardness of the sample but also influences the wear performance. Therefore, the surface microstructure of wheel-rail samples were analyzed in detail, as displayed in Figure 7, Figure 8, Figure 9 and Figure 10. Figure 7 shows the OM micrographs of wheel samples under different contact stress conditions. Under low contact stress condition (700 MPa), the thickness of the plastic deformation layer was thin, which was only about 60 μm. As the contact stress increased, the thickness of the plastic deformation layer was increased gradually. Under a contact stress of 1200 MPa, the thickness of the plastic deformation layer reached up to 120 μm. 

The evolution of the microstructure of wheel samples at different depths from the surface under a contact stress of 1200 MPa was characterized by the use of SEM, as shown in Figure 8. At depths of 150–160 μm from the surface, there was no obvious plastic deformation in pearlite, and the shape of grains in proeutectoid ferrite was equiaxed. At a depth of 80–90 μm below the surface, the shape of the grains in proeutectoid ferrite was changed from equiaxed toward lamellae as a consequence of shear stress, while the cementite was still lamellar. At a depth of 30–40 μm below the surface, the lamellar grains in proeutectoid ferrite were refined further, and a part of lamellar cementite was broken into particles. At depths of 0–10 μm, the lamellar cementite was broken into particles completely.

The OM micrographs of rail samples under different contact stress conditions are presented in Figure 9. The evolution of the plastic deformation layer of rail samples is similar to that of the wheel samples. With the increase of contact stress, the thickness of the plastic deformation layer was increased gradually. It is similar to the result of W Zhong et al [8]. Under contact stress of 700 MPa and 900 MPa, the thickness of the plastic deformation layer of rail samples was thinner as compared with wheel samples. That is because the yield strength of rail samples (880 MPa) was higher than that of wheel steel (615 MPa), thereby yielding a stronger resistance of plastic deformation in rail samples. However, under high contact stress condition (1200 MPa), the thickness of the plastic deformation layer of rail samples was increased obviously, which reached to around 110 μm. Its thickness was nearly the same as that of wheel samples. There was more proeutectoid ferrite in wheel samples. The rate of the plastic of wheel samples was faster than that of rail samples. As a result, the plastic deformation of wheel samples was faster to reach up to the steady state (the wear loss and the increase of plastic deformation of samples are balanced [23]). The thickness of the plastic deformation layer of wheel samples was hardly changed in the steady state under this contact stress condition. High contact stress (1200 MPa) was far above the yield strength of rail samples (880 MPa). Therefore, it could also lead to obvious plastic deformation of rail samples to form the thicker plastic deformation layer. 

Figure 10 is the evolution of the microstructure of rail samples along the depth from the surface under a contact stress of 1200 MPa. At depths of 150–160 μm from the surface, no plastic deformation occurred in pearlite. At depths of 80–90 μm from the surface, the cementite in pearlite was still lamellar. However, the plastic deformation had occurred in pearlite. At depths of 30–40 μm from the surface, the plastic deformation of pearlite was obvious. An amount of lamellar cementite was broken into particles. The direction of ferrite grains tended to be parallel with the rolling direction. At depths of 0–10 μm from the surface, the lamellar cementite was broken into particles completely, and a part of cementite was dissolved at the surface. 

### 3.4. Surface Wear Morphology

**Figure 11:**
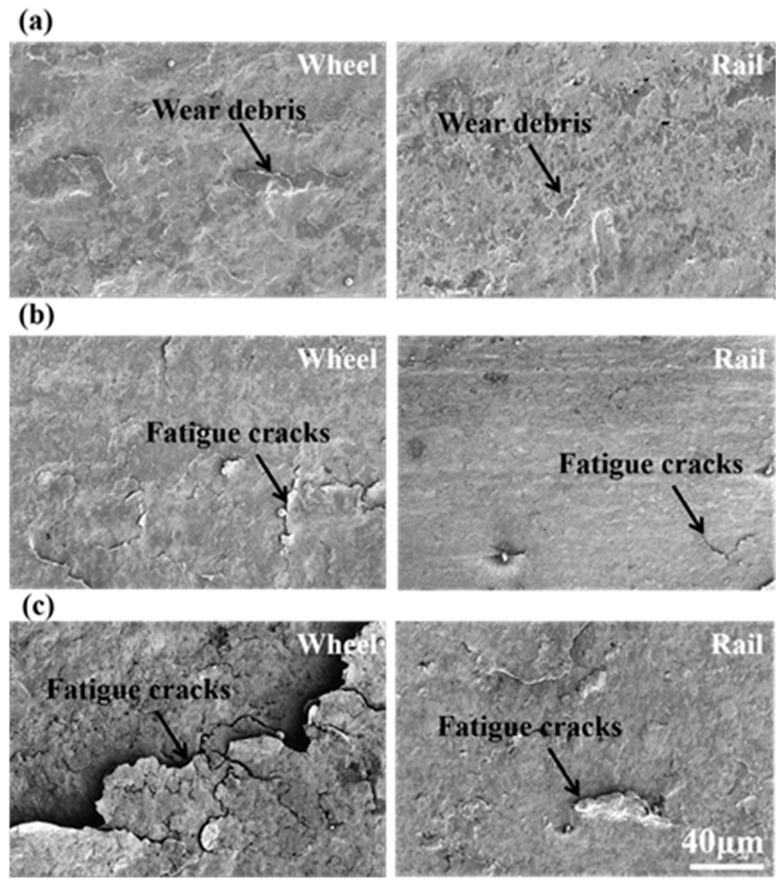
The surface wear morphology of wheel and rail samples under different contact stress conditions: (**a**) 700 MPa; (**b**) 900 MPa and (**c**) 1200 MPa.

**Figure 12:**
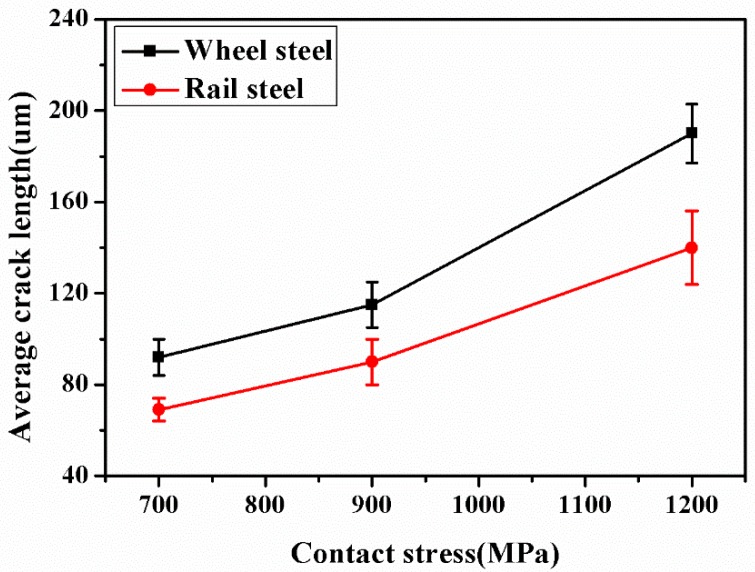
The average crack length of wheel and rail samples under different contact stress conditions.

Figure 11 is the surface wear morphology of wheel-rail samples under different contact stress conditions. Under a contact stress of 700 MPa, the surface of wheel-rail samples formed an amount of wear debris. There was no fatigue wear cracks on the surface of samples. The wear mechanism of wheel-rail samples was adhesive wear. Under a contact stress of 900 MPa, the surface of the wheel-rail samples surface formed a small amount of fatigue wear cracks. The fatigue wear cracks of wheel samples were severer than that of rail samples. The wear mechanism was the combination of adhesive wear and fatigue wear. Under a contact stress of 1200 MPa, the wheel-rail samples surface formed a large amount of fatigue wear cracks. The fatigue wear cracks of wheel samples were severer than that of rail samples. Therefore, the wear mechanism of wheel-rail samples was fatigue wear. The fatigue wear cracks initiate at the surface and propagate along the interface of the fiber deformation layer [24]. It can be seen from Figure 12 that the average crack length of samples was increased as the contact stress increase. Moreover, the average crack length of wheel samples was longer than that of rail samples. 

## 4. Discussion

**Figure 13:**
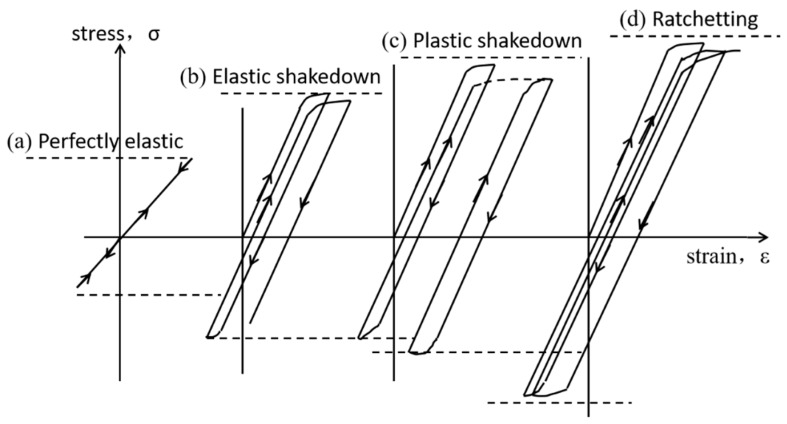
The schematic diagram of the stress and strain of materials during rolling operation [25].

Figure 13 is the schematic diagram of the stress and strain of material during rolling operation. At low stress, the material is in an elastic scope and the material cannot produce plastic deformation. With an increase of stress, the material will reach the elastic limit and plastic strain will occur. According to the Coffin–Manson law [25]:

(4) $$C=\left(\frac{\Delta \varepsilon_{p}}{2}\right)N_{f}^{n}.$$

In formulation, *C* is the fatigue ductility, Δ*ε_p_* is the ratchet rate, *N_f_* is the cycles to ratcheting failure and *n* is the fatigue exponent (n = 1/2). When the materials produce the plastic strain at the critic stress level, the accumulation of plastic strain of material was increased with the increase of cycles. The fatigue ductility (C) was increased with the increase of cycles. After some cycles, the fatigue ductility (C) will exceed the ductility limit, i.e., ratcheting failure [26]. The ratcheting failure is the process that material accumulates the plastic strain reach to the ductility limit to lead to the failure of materials [24,27]. 

**Figure 14:**
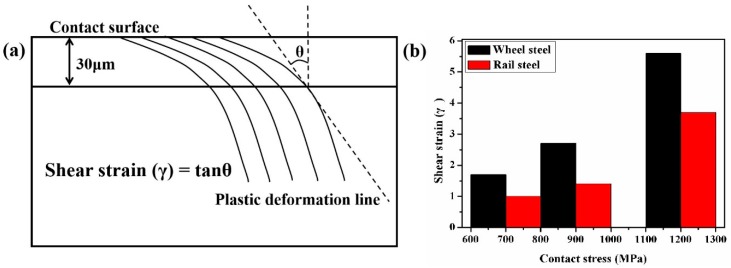
The variation of the shear strain of wheel and rail samples. (**a**) The schematic diagram of the shear strain measurement technique [28] and (**b**) the variation of shear strain values.

Figure 14a is the schematic diagram of the shear strain measurement technique. The *θ* is acquired by measuring the included angle between plastic flow at the depth of 30 μm from the surface and vertical rolling direction. The shear strain *γ* is calculated by the following equation:

(5) γ=tan(θ).

Under a contact stress of 700 MPa, the shear strain of samples was lower, as displayed in Figure 14b. Therefore, the degree of plastic deformation and surface hardness of samples was smaller under this contact stress condition. Therefore, the plastic deformation of samples was not reaching the ratcheting failure. Therefore, under this contact stress condition, the wear mechanism of samples was adhesive wear. Under a contact stress of 900 MPa, the further increase of contact stress contributed to the formation of a small amount of fatigue wear cracks at the sample surface. Under a contact stress of 1200 MPa, the shear strain of samples increased sharply. The shear strain of wheel and rail samples was 5.5 and 3.5, respectively. The thickness of the plastic deformation layer and surface hardness was at its maximum (Figure 6, Figure 7 and Figure 9). The plastic strain of wheel and rail samples was increased with the increase of cycles. When the accumulation of the plastic strain reached the ductility limit it produced a large amount of fatigue wear cracks at surface samples. Therefore, the wear mechanism was fatigue wear under this contact stress condition. 

The wear rate is calculated by the following equation [29,30,31]:

(6) $$K=\frac{V}{F_{N}\times S},$$

where the *K* is the wear rate, *F_N_* is the applied load (*F_N_* is the linear relation with contact stress), *V* is the wear volume (*V* is the linear relation with wear loss (Figure 5)) and *S* is the rolling distance. Therefore, the wear rate (K) is the linear relationship with the ratio of wear loss to contact stress. Under a contact stress of 700 MPa, the wear mechanism was adhesive wear, the small wear debris were spalling, as shown in Figure 11a. The wear rate (K) of wheel-rail samples was low. Although the surface hardness of wheel-rail samples was maximum under a contact stress of 1200 MPa, the wear rate (K) of wheel-rail samples was also at its maximum. The reason should be that the wear mode of wheel and rail samples was the layer by layer flaking of fatigue wear cracks under a contact stress of 1200 MPa, as shown in Figure 11c. After the surface fatigue wear cracks are flaked, the surface of samples will form the fatigue wear cracks rapidly under the high contact stress condition. During the rolling wear process, a large amount of fatigue wear cracks were flaked gradually according to cycles to cause the wear rate of samples being high under a contact stress of 1200 MPa. Therefore, fatigue wear was faster to lead to the failure of wheel-rail samples than that of adhesive wear. 

The fatigue wear cracks of wheel samples were severer than that of rail samples. The matrix hardness of wheel samples was lower than that of rail samples. During the rolling wear process, the increase of the plastic strain of wheel samples was faster than that of rail samples. As a result, the accumulation of the plastic strain of wheel samples faster reached ratcheting failure. Another reason is that there was more proeutectoid ferrite in wheel samples than that in rail samples, as displayed in Figure 1. When plastic deformation occurred in hypo eutectoid pearlite steel, pearlite colonies and adjacent proeutectoid ferrite could not deform conformably. The fatigue wear cracks will primarily initiate at the interface between pearlite colonies and adjacent proeutectoid ferrite [6]. The hardening ratio of proeutectoid ferrite is two times higher than that of pearlite [32]. The proeutectoid ferrite is also the site that causes the initiation of fatigue wear cracks [33]. Therefore, the fatigue wear cracks of wheel samples were severer than that of rail samples.

## 5. Conclusions

In this paper, the surface microstructure and wear property in the D2/U71Mn wheel-rail material under different contact stress conditions were studied by using a GPM-40 wear machine, the following conclusions were drawn as:

(1) Under different contact stress conditions, the variation of traction coefficient presented similar trends according to cycles. Under the contact stress of 900 MPa, the traction coefficient value was at its maximum. The polygonization wear of wheel samples led to the existence of the slip ratio between wheel and rail samples. The ship ratio caused the increase of the traction coefficient. Under a contact stress of 1200 MPa, the contact surfaces of wheel-rail samples were smooth and the traction coefficient value was at its minimum.

(2) The degree of plastic deformation (surface hardness and thickness of plastic deformation layer) was increased with the increase of contact stress. Under the high contact stress condition (1200 MPa), the severe plastic deformation caused the formation of fatigue wear of wheel-rail samples. 

(3) Under a contact stress of 700 MPa, the wear mechanism of wheel-rail samples was adhesive wear. The wear rate was low. With the increase of contact stress, the fatigue cracks were severe gradually. Under a contact stress of 1200 MPa, the wear mechanism of wheel-rail samples transformed into fatigue wear. The wear rate was high. Fatigue wear was faster to lead to the failure of wheel-rail samples than that of adhesive wear. The contact stress of D2/U71Mn rail-wheel material should be no more than 1200 MPa to inhibit the formation of fatigue wear. 

(4) The fatigue wear cracks of wheel samples were severer than that of rail samples. The increase of the plastic strain of wheel samples was faster than that of rail samples. As a result, the accumulation of the plastic strain of wheel samples faster reached ratcheting failure. There was more proeutectoid ferrite in wheel samples. The proeutectoid ferrite also was the site that caused the initiation of fatigue wear cracks.

## Figures and Tables

**Table 1:** Chemical components of the wheel-rail samples (%).

Samples	C	Si	Mn	S	P
D2	0.50–0.56	0.90–1.10	0.90–1.10	≤0.010	≤0.015
U71Mn	0.65–0.77	0.15–0.35	1.00–1.40	≤0.03	≤0.03
